# The hypolipidemic mechanism of chrysanthemum flavonoids and its main components, luteolin and luteoloside, based on the gene expression profile

**DOI:** 10.3389/fnut.2022.952588

**Published:** 2022-09-06

**Authors:** Jihan Sun, Zhaodan Wang, Chen Lin, Hui Xia, Ligang Yang, Shaokang Wang, Guiju Sun

**Affiliations:** ^1^Key Laboratory of Environmental Medicine and Engineering of Ministry of Education, Department of Nutrition and Food Hygiene, School of Public Health, Southeast University, Nanjing, China; ^2^College of Biology and Food Engineering, Technology Research Center of Characteristic Biological Resources in Northeast of Chongqing, Chongqing Three Gorges University, Chongqing, China

**Keywords:** chrysanthemum flavonoids, luteolin, luteoloside, hyperlipidemia, mechanism

## Abstract

In this study, the following four groups of mice with hyperlipidemia were involved: the model control group (MC), the Chrysanthemum flavonoids group (CF), the luteolin group, and the luteoloside group. The whole gene expression profile was detected in the liver tissues of each group. Differential genes significantly enriched in the biological process of gene ontology (GO) items and Kyoto Encyclopedia of Genes and Genomes (KEGG) were selected, and 4 differential genes related to lipid metabolism were selected for further real-time quantitative PCR verification. Compared with the MC, 41 differential genes such as Sqle, Gck, and Idi1 were screened in the CF intervention group; 68 differential genes such as Acsl3, Cyp7a1, and Lpin1 were screened in the luteolin intervention group (CF); and 51 differential genes such as Acaca, Cyp7a1, and Lpin1 were screened in the luteoloside group. The mechanism of CF to improve hyperlipidemia is very complex, mainly involving biological processes such as cholesterol and fatty acid metabolism and glycolysis, luteolin mainly involves the synthesis and transport of cholesterol, and luteoloside mainly involves fatty acid metabolism. The functional pathways of CF may not be completely the same as luteolin and luteoloside, and further study is needed on the mechanism of action of other components.

## Introduction

Hyperlipidemia refers to one or more metabolic diseases with abnormal blood lipids (1). Most patients with hyperlipidemia have no obvious clinical symptoms, but it is a major risk factor for cardiovascular and cerebrovascular diseases (2). The 2015 Survey in China showed that the rate of dyslipidemia among adults was 40.4%. In view of the limitations of clinical lipid-lowering drugs, several studies are focusing on the lipid-lowering efficacy and application of natural products. Phytochemicals can be further developed as a potential new natural, safe, and efficient lipid-lowering drugs because of their cheap, safe, and efficient characteristics. The known phytochemicals with lipid-lowering effects are phytosterols, phenols, saponins, alkaloids, organic sulfides, and lectins (3).

At present, the research on the lipid-lowering activity of flavonoids is more and more extensive and in-depth. Foodborne flavonoids can improve lipid metabolism and prevent cardiovascular disease (4–6). The lipid-lowering activity of flavonoids may be achieved by affecting a variety of lipid metabolism pathways in the intestine and the liver, such as inhibiting intestinal absorption of lipids and increasing lipid excretion (7, 8), activating adenylate-activated protein kinase (AMPK) so as to promote lipid catabolism or inhibit lipid synthesis (9), and regulating microRNAs (miRNAs) with the function of regulating lipid metabolism (10).

Chrysanthemum has been used mainly in traditional herbal teas and beverages, and various studies have shown that it has biological activities to lower blood lipids and fight obesity. Chrysanthemum extract alleviated the fatty liver in mice by the PPARα-mediated pathway (11). After enzymatic treatment, lipid accumulation and production in obese male mice induced by a high-fat diet were reduced (12). *In vitro*, it inhibited adipogenesis by inhibiting mitosis in the early differentiation of 3T3-L1 cells (13). *In vivo* and *in vitro* experiments have shown that chrysanthemum can activate the AMPK pathway to inhibit fat production (14, 15).

In our previous study, we compared the effects of Chrysanthemum flavonoids (CF), luteolin, and luteoloside, on improving blood lipid and liver steatosis, and found that the preliminary mechanism may be related to antioxidant levels and enzymes related to regulating fatty acid, cholesterol, and triglyceride metabolism in the liver (16). In this study, differential genes are screened out by a gene chip, and the molecular mechanism of their function is further discussed from the biological process and signal pathway.

## Materials and methods

### Materials

The animal ethics approval was 2015ZDSYLL004.0. We selected liver tissues from the pre-study animals (16) for whole gene expression profiling to explore the mechanism of lipid lowering, with three samples from each group. The Agilent SurePrint G3 Rat GE V2.0 Microarray (8*60K,Design ID:074036) was used in this experiment, provided by Shanghai Ouyi Biomedical Technology Co., Ltd., the chip contains 45,738 probes.

### Extraction of total ribonucleic acid from liver tissue

Total ribonucleic acid (RNA) of liver tissue was extracted from 100 mg liver tissue using mirVana™ RNA Isolation Kit, AM1561 Kit. The total RNA was preserved at -80°C. NanoDrop ND-2000 (Thermo Fisher Scientific, China) was used to detect the absorbance of the extracted RNA at 260 and 280 nm, and the concentration of the total RNA was calculated. Agilent Bioanalyzer 2100 (Agilent, United States) was used to carry out the integrity test (with 2100RIN ≥ 7 and 28/18 s ≥ 0.7 as the qualified standard) before the gene chip test can be carried out. Three samples from each group were randomly selected for follow-up chip experiments.

### Expression gene profile detection

An amount of 0.2 μg of total RNA was taken, and 2.5 μl of deionized water and 2.8 μl of the reaction mixture (2 μl of Spike Mix and 0.8 μl of T7 Promoter Prime) were added. After mixing using centrifugation, denaturing for 10 min at 65°C and ice bath for 5 min were performed. A volume of 2 μl of 5 × First-Strand Buffer (preheat at 80°C for 3 min) was taken, 1 μl of 0.1 m dithiothreitol (DTT), 0.5 μl of 10 mM deoxyribonucleoside triphosphate (dNTP) mix, and 1.2 μl of AffinityScript Rnase BLock Mix were added, the sample was mixed well, and then the RNA was added. Reaction PCR was performed after mixing and centrifugation (40°C for 2 h, 70°C for 15 min, and ice bath for 5 min). All reagents were obtained from Agilent Low Input Fast Amplifier Labeling kit (Agilent, United States).

### Synthesis and purification of fluorescently labeled cDNA

Notably, 0.75 μl of H_2_O, 3.2 μl of 5 × transcription buffer, 0.6 μl of 0.1 m DTT, 1 μl of NTP mix, 0.21 μl of Cy3-CT (to avoid light), and 0.24 μl of T7 RNA polymerase blend were added into the sample tube and allowed to react for 2 h at 40°C. Then, 84 μl of enzyme-free water, 350 μl of BufferRLT, and 250 μl of anhydrous ethanol were added and the sample was centrifuged for 30 s (4°C, 2,500 rpm) to discard the filtrate. Next, 500 μl of buffer RPE was added to wash the filtrate two times. After washing, 30 μl of non-enzyme water was added, the sample was centrifuged (4°C, 10,000 *g*) for 1 min, and then the filtrate was collected. All reagents were obtained from Agilent Low Input Fast Amplifier Labeling kit (Agilent, United States).

### Determination of cRNA concentration

The concentration of purified RNA was determined by NanoDrop ND-2000 (Thermo Fisher Scientific, China). After adjusting to zero with water, 1 μl of the sample was used to determine and record the concentration of cRNA and the content of fluorescence. The specific calculation formula was given as follows: cRNA content (μg) = cRNA concentration (ng/μl) × 30 μl/1,000; Cy3-incorporation rate (pmol/μg) = Cy3-concentration/cRNA concentration (ng/μl) × 1,000.

### Sample fragmentation and chip hybridization of cRNA

The hybrid furnace was preheated at 65°C, and a fragmentation mixture was added and then placed in the ice bath for 1 min. cRNA from Fragmentation Mix and 2 × GE Hyb Hi-RPM Buffer were added. The cover was removed from the hybrid rack, and samples were added to each hole of the chip. The required hybrid chip was taken out, the Agilent marker was covered face down horizontally, the hybridization device was assembled and tightened, and the chip was turned clockwise three times to fully combine the hybrid liquid with the probe. The hybrid frame was placed in a hybrid furnace for 17 h for rolling hybridization under the condition of 65°C and 10 rpm.

### Chip washing and scanning

The chip was washed three times in the washing cylinder. The chip was put forward slowly to ensure that there were no residual water droplets, and the chip was carefully placed face up into the scanning rack. Agilent scanner G2505C (Agilent, United States) was used to scan the chip.

### Screening of differential genes

The fluorescence signal values of the images were processed by Feature Extraction and standardized by GeneSpring 13.1 (Agilent, United States). Taking the difference significance *P*-value and difference multiple (fold change, Fc) value as reference, the differential genes were screened according to the absolute value of Fc ≥ 2 and *P* ≤ 0.05. The functions and pathways of annotated differential genes were analyzed.

### Real-time fluorescence quantitative PCR verification

Quantification was performed with a two-step reaction process, namely, reverse transcription (RT) and PCR. Each RT reaction had two steps. The first step was to add 0.5 μg of RNA, 2 μl of 4 × gDNA wiper mix, and nuclease-free H_2_O to 8 μl. The sample was allowed to react for 2 min at 42°C. The second step was to add 2 μl of 5 × HiScript II Q RT SuperMix IIa. The sample was allowed to react for 10 min at 25°C, 30 min at 50°C, and 5 min at 85°C. The 10 μl of RT reaction mix was then diluted 10 times in nuclease-free H_2_O and held at -20°C. Real-time PCR was performed with a 10-μl PCR reaction mixture that included 1 μl of cDNA, 5 μl of 2 × QuantiFast^®^ SYBR^®^ Green PCR Master Mix (Qiagen, Germany), 0.2 μl of forward primer, 0.2 μl of reverse primer, and 3.6 μl of nuclease-free H_2_O. Reactions were incubated in a 384-well optical plate (Roche, Swiss) at 95°C for 5 min, followed by 40 cycles of 95°C for 10 s and 60°C for 30 s. The primer sequences (Table 1) were designed in the laboratory and synthesized by Generay Biotech (Generay, PRC). The expression levels of mRNAs were normalized to ACTB and were calculated using the 2^–ΔΔCt^ method [Livak and Schmittgen, (17)].

**TABLE 1:** Primer sequences of qPCR verified genes.

Gene	Upstream primer	Downstream primers	Length (bp)
Gpam	TACGCTGAGAGTGCCACATA	GTTCTAAGACAGACGCTCG	106
Acsl3	CCTCCTCCAGTTTGCTTTG	ATCACTGCTTCCCCAGTA	81
Cyp7a1	CTTAGAACAAGTTTGATGACTC	CGTGAAACCCATCATTCTGT	100
Lpin1	GAGCAGGATGGACTGTTACT	GCCGTTCCGGTGAATTATG	112
Sqle	AAGAATGATTGTTTCCACAAAT	TTTATTGGCATGTCCCAATGA	85
Gck	GCCTCACTCTGCACTATTCA	GTGGTCTCTTGGAGGGACA	97
Gpam	TACGCTGAGAGTGCCACATA	GCTCAGTTCTAAGACAGACG	111
Acaca	CGAGATTTCACTGTGGCTT	GCAATACCATTGTTGGCGATA	95
Angptl4	GAGCCCTGGATACACTCAAT	TGTTGTGAGCTGTGCCTT	85

### Statistical analysis

The data were analyzed using SPSS16.0 (IBM, United States), which was expressed as mean ± standard deviation, and a *P-*value of < 0.05 was considered statistically significant.

## Results

### Chip hybrid scan image

**FIGURE 1:**
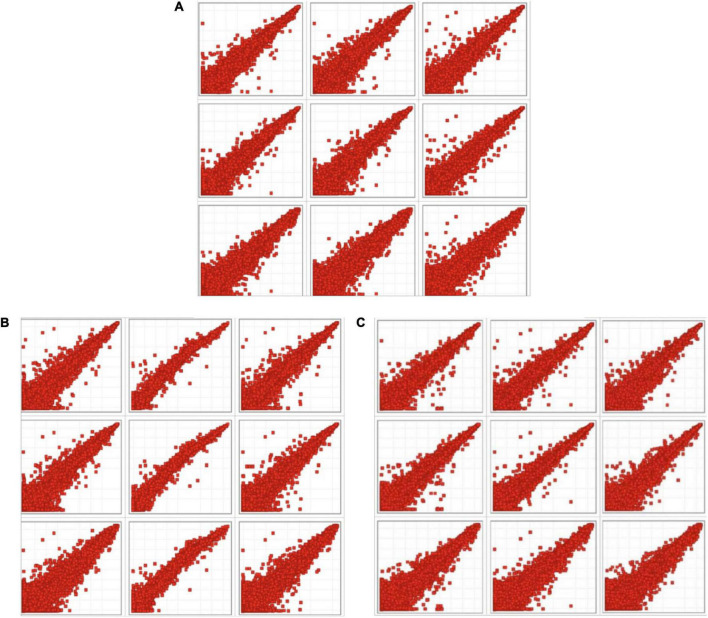
Scatter plot of intervention groups compared with the model control group. **(A)** Scatter plot of fluorescence signal values in chrysanthemum flavonoids intervention group (CF) and model control group. **(B)** Scatter plot of fluorescence signal values in luteolin intervention group and model control group. **(C)** Scatter plot of fluorescence signal values in luteoloside intervention group and model control group.

In the scatter plot (Figure 1), each point represents a probe, the horizontal axis represents the sample fluorescence signal value of the intervention group, and the longitudinal axis represents the sample fluorescence signal value of the model control group (MC), which on the y = x line indicate that the signal values of the two samples are equal (multiple of difference Fc = 1). The point that fall above the line (y = x) represents upregulation and that fall below the line represents downregulation.

### Screening of differential genes between intervention group and model control group

**FIGURE 2:**
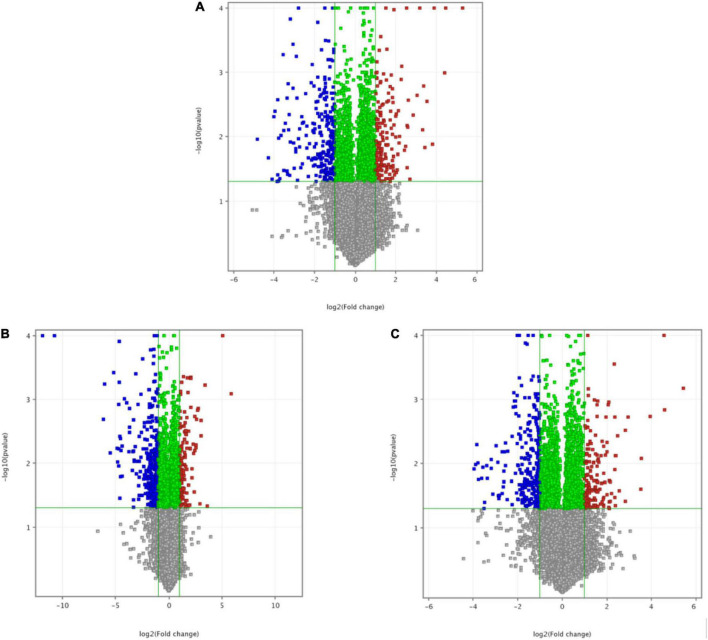
A differential gene volcano map of intervention groups compared with the model control group. **(A)** Volcano map of model control group and chrysanthemum flavonoids intervention group. **(B)** Volcano map of model control group and luteolin intervention group. **(C)** Volcano map of model control group and luteoloside intervention group.

Figure 2 shows a differential gene volcano map of MC and intervention groups, the horizontal axis represents the logarithm of the Fc based on 2, and the vertical axis represents the negative logarithm of the *P*-value with the base of 10. Red represents significantly upregulated genes, and blue represents significantly downregulated genes. Compared with the MC, there were 427 differential genes in the CF intervention group (260 downregulated genes and 167 upregulated genes significantly), 451 differential genes in the luteolin intervention group (335 downregulated genes and 116 upregulated genes significantly), and 420 differential genes in the luteoloside intervention group (260 downregulated genes and 160 upregulated genes significantly).

### GO analysis of differential genes between intervention group and model control group

**FIGURE 3:**
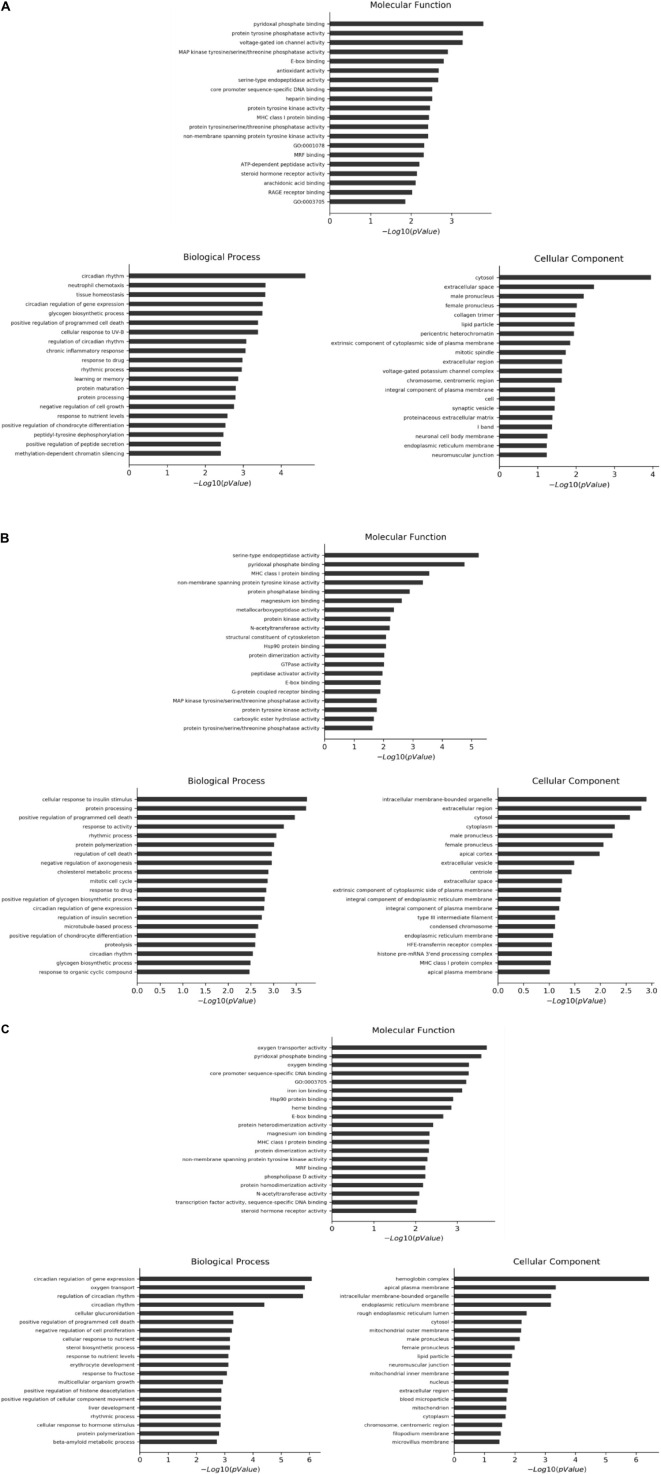
First 20 GO items with significant enrichment of differential genes in each plate. **(A)** CF group vs. model control group. **(B)** Luteolin group vs. model control group. **(C)** Luteoloside group vs. model control group.

The selected differential genes were functionally annotated by the GO database, which can be divided into three parts. *P* ≤ 0.05 indicates that the differential genes are significantly enriched in the GO entry. Table 2 shows the number of significantly enriched items in the three GO plates in different intervention groups compared with the MC. The first 20 GO items with significant enrichment of differential genes in each plate are shown in Figure 3.

**TABLE 2:** Functional classification of differentially expressed genes.

GO analyze	Biological process	Molecular function	Cellular component
**CF group vs. model control group**			
Annotated	1,067	304	193
Significant enrichment	121	32	9
**luteolin group vs. model control group**			
Annotated	1,190	323	196
Significant enrichment	159	52	23
**Luteoloside group vs. model control group**			
Annotated	1,133	322	212
Significant enrichment	131	37	17

### KEGG analysis of differential genes between intervention group and model control group

**FIGURE 4:**
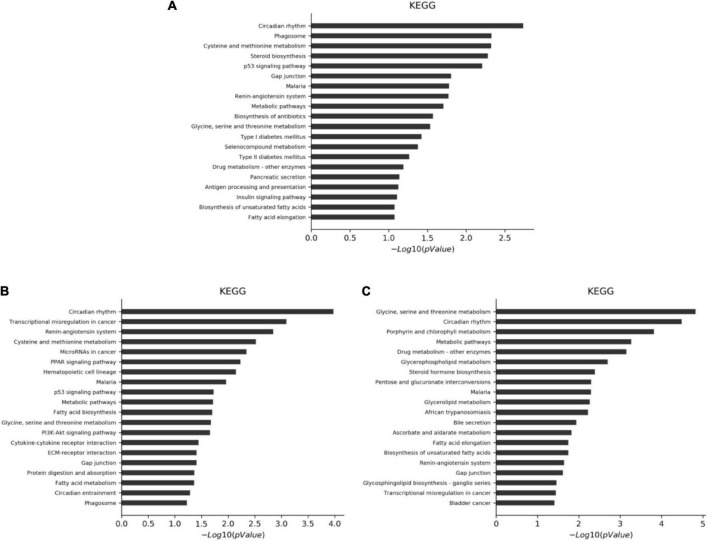
KEGG analysis of differential gene significant enrichment pathways (top 20). **(A)** CF group vs. model control group. **(B)** Luteolin group vs. model control group. **(C)** Luteoloside group vs. model control group.

The differential genes were analyzed by KEGG. A *P*-value of < 0.05 indicates a significant enrichment of differential genes. The results showed that there were 186 annotable items in the CF group compared with the MC, among which 13 were significantly enriched. There were 187 annotable items in the luteolin group compared with the MC, among which 26 were significantly enriched. There were 180 annotable items in the luteoloside group compared with the MC, among which 18 were significantly enriched. The top 20 significantly enriched pathways in each group are shown in Figure 4.

### Comprehensive analysis and verification gene selection

**FIGURE 5:**
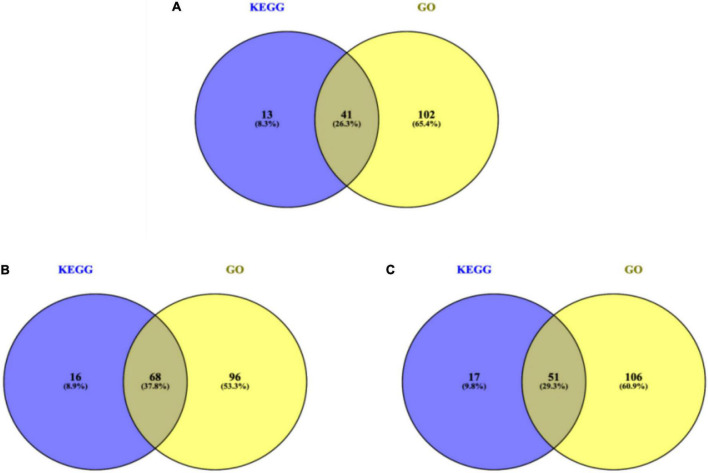
The number of genes in each intervention group. **(A)** CF group vs. model control group. **(B)** Luteolin group vs. model control group. **(C)** Luteoloside group vs. model control group.

Differential genes significantly enriched in the biological process of GO items and KEGG were selected (the number of genes in each intervention group is shown in Figure 5), and 4 differential genes related to lipid metabolism were selected for further real-time quantitative PCR verification. KEGG items with a significant enrichment and GO biological process items involved in chip results are summarized in Table 3.

**TABLE 3:** Differential genes both significantly enriched in KEGG and GO biological process items.

Group	GeneSymbol	KEGG	GO
**CF group vs. model control group**	Sqle	rno00100 steroid biosynthesis rno01100 metabolic pathways rno01130 biosynthesis of antibiotics	GO:0008203 metabolic pathways GO:0010033 response to organic substance
	Gck	rno01100 metabolic pathways rno01130 biosynthesis of antibiotics	GO:0032869 cellular response to insulin stimulus GO:0045725 positive regulation of glycogen biosynthetic process GO:0050796 regulation of insulin secretion GO:0005978 glycogen biosynthetic process GO:0055088 lipid homeostasis GO:0009749 response to glucose GO:0032024 positive regulation of insulin secretion
	Gpam	rno01100 metabolic pathways	GO:0032869 cellular response to insulin stimulus GO:0014823 response to activity GO:0055089 fatty acid homeostasis GO:0009749 response to glucose GO:0006641 triglyceride metabolic process GO:0010867 positive regulation of triglyceride biosynthetic process GO:0006637 acyl-CoA metabolic process GO:0006631 fatty acid metabolic process
	Idi1	rno01100 metabolic pathways rno01130 biosynthesis of antibiotics	GO:0006695 cholesterol biosynthetic process
**Luteolin group vs. model control group**	Acsl3	rno01100 metabolic pathways	GO:0014070 response to organic cyclic compound GO:0001676 long-chain fatty acid metabolic process
	Cyp7a1	rno01100 metabolic pathways rno00140 steroid hormone biosynthesis rno04976 bile secretion	GO:0071333 cellular response to glucose stimulus GO:0071397 cellular response to cholesterol GO:0055114 oxidation-reduction process
	Gpam	rno01100 metabolic pathways rno00564 glycerophospholipid metabolism rno00561 glycerolipid metabolism	GO:0031667 response to nutrient levels GO:0009750 response to fructose GO:0032869 cellular response to insulin stimulus GO:0006641 triglyceride metabolic process GO:0006637 acyl-CoA metabolic process GO:0014823 response to activity GO:0006631 fatty acid metabolic process GO:0055089 fatty acid homeostasis GO:0019432 triglyceride biosynthetic process GO:0046686 response to cadmium ion GO:0042104 positive regulation of activated T cell proliferation
	Lpin1	rno01100 metabolic pathways rno00564 glycerophospholipid metabolism rno00561 glycerolipid metabolism	GO:0031065 positive regulation of histone deacetylation GO:0032869 cellular response to insulin stimulus GO:0019432 triglyceride biosynthetic process GO:0006470 protein dephosphorylation GO:0000122 negative regulation of transcription from RNA polymerase II promoter
**Luteoloside group vs. model control group**	Acaca	rno01100 metabolic pathways rno00061 fatty acid biosynthesis rno01212 fatty acid metabolism	GO:0001894 tissue homeostasis GO:0042493 response to drug GO:0014070 response to organic cyclic compound GO:0055088 lipid homeostasis GO:0006629 lipid metabolic process
	Angptl4	rno03320 PPAR signaling pathway	GO:0019216 regulation of lipid metabolic process GO:0051260 protein homooligomerization GO:0043066 negative regulation of apoptotic process GO:0001666 response to hypoxia
	Cyp7a1	rno03320 PPAR signaling pathway rno01100 metabolic pathways	GO:0071397 cellular response to cholesterol
	Lpin1	rno01100 metabolic pathways	GO:0000122 negative regulation of transcription from RNA polymerase II promoter GO:0031065 positive regulation of histone deacetylation GO:0045598 regulation of fat cell differentiation GO:0006629 lipid metabolic process GO:0031100 organ regeneration

### RT-PCR verification results of the intervention group and the model control group

**FIGURE 6:**
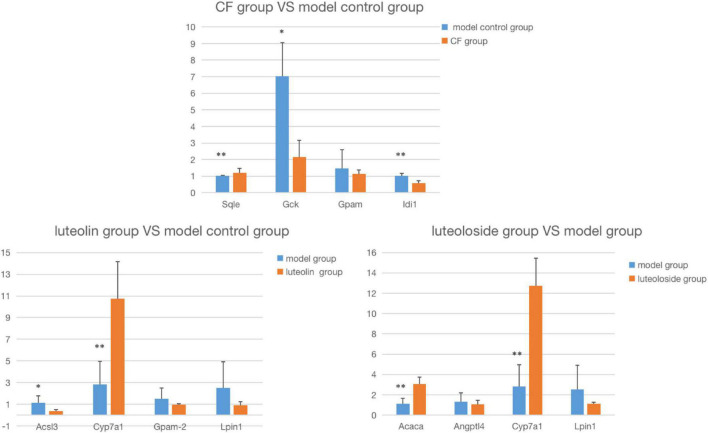
The comparison between the RT-PCR and gene chip results. Compared with the model control group: *P < 0.05; **P < 0.01.

The comparison between the RT-PCR and gene chip results is shown in Figure 6. The expression trend is completely the same, suggesting that the data of the gene chip are reliable. As shown in Table 4, compared with the MC, in the luteolin intervention group, the expression of Acsl3 and Cyp7a1 was significantly upregulated, and the expression of **Lpin1** was significantly downregulated, while there was no statistical difference in Gpam expression downregulation; in the CF intervention group, the expression of Sqle, Gck, and Idi1 was statistically and significantly downregulated, while there was no statistical difference in Gpam expression downregulation; in the luteoloside intervention group, the expression of Acaca and Cyp7a1 was significantly upregulated and the expression of **Lpin1** was significantly downregulated, while there was no statistical difference in Angptl4 expression downregulation.

**TABLE 4:** Gene expression verified by real-time PCR.

CF group vs. model control group	Sqle	Gck	Gpam	Idi1
Model control group	1.21 ± 0.27	7.04 ± 5.71	1.48 ± 0.97	1.01 ± 0.15
CF group	1.00 ± 0.04*	2.16 ± 0.87**	1.15 ± 0.21	0.57 ± 0.13*
Expression	0.83	0.31	0.78	0.56
PCR differential multiple	−1.21	−3.26	−1.28	−1.79
Gene chip differential multiple	−2.63	−5.22	−2.50	−2.91
**Luteolin group vs. model control group**	**Acsl3**	**Cyp7a1**	**Gpam**	**Lpin1**
Model control group	1.16 ± 0.64	2.84 ± 2.12	1.51 ± 0.99	2.52 ± 2.40
Luteolin group	0.36 ± 0.18**	10.74 ± 3.42**	0.97 ± 0.11	0.91 ± 0.32*
Expression	0.31	3.78	0.65	0.36
PCR differential multiple	−3.22	3.78	−1.55	−2.77
Gene chip differential multiple	−2.97	2.75	−3.46	−4.31
**Luteoloside group vs. model control group**	**Acaca**	**Angptl4**	**Cyp7a1**	**Lpin1**
Model control group	1.13 ± 0.52	1.32 ± 0.85	2.84 ± 2.12	2.52 ± 2.40
Luteoloside group	3.08 ± 0.67**	1.06 ± 0.42	12.73 ± 2.73**	1.10 ± 0.17**
Expression	2.72	0.80	4.48	0.44
PCR differential multiple	2.72	−1.24	4.48	−2.29
Gene chip differential multiple	2.06	−2.09	2.09	−3.50

Expression was 2^–ΔΔ*Ct*^ ratio of intervention group and model control group: Expression < 1, gene expression downregulated; Expression > 1, gene expression upregulated; Compared with model control group: *P < 0.05; **P < 0.01.

## Discussion

The results suggest that compared with the normal control group, mice with hyperlipidemia have different biological processes that may involve monounsaturated fatty acid biosynthesis processes, cellular response to insulin stimulation, steroid decomposition processes, and response to fatty acids. The involved signaling pathways include the PPAR signaling pathway, biosynthesis of unsaturated fatty acids, and fatty acid metabolism.

In this experiment, differential genes in the intervention group and MC were screened and 4 genes related to lipid metabolism were selected for RT-PCR verification. Compared with the MC, 41 genes were both significantly enriched in GO and KEGG analyses in the CF intervention group. The results of gene verification are consistent with the trend of gene chip. Sqle, Gck, and Idi1 were significantly downregulated. Squalene cyclooxygenase (Sqle) is a rate-limiting and first oxygenation enzyme of cholesterol biosynthesis and is considered a proto-oncogene. Sqle induced the development of non-alcoholic fatty liver disease (NAFLD) by inducing cholesterol biosynthesis and Sqle/CA3-axis-mediated adipogenesis in Sqle overexpression transgenic mice (18). Meanwhile, SQLE silencing can limit the occurrence of liver tumors induced by NAFLD (19). The results of our previous animal experiments also support the idea that the intervention group of CF improved the hepatic steatosis of hyperlipidemic rats (16). Gluckinase (GCK) is a key enzyme regulating insulin release and the first step in catalyzing glycolysis in the liver (20). Its genetic polymorphisms are associated with susceptibility to gestational diabetes. Studies have shown that the GCK RS1799884 mutation is associated with a higher incidence of gestational diabetes mellitus (GDM) in the Chinese population (21). Its expression is also associated with hyperlipidemia, and the molecular mechanism of the regulation of hyperlipidemia by instant dark tea (IDT) has been demonstrated in rats fed with a high-fat diet and is related to the significant influence on the expression of glycoly-related genes such as GCK (22). Isoprene diphosphate isomerase (IDI) is involved in the biosynthesis of isoprene-like cholesterol, and there are two subtypes in humans, namely, IDI1 and IDI2 (23). IDI1 plays a regulatory role in atrial lipotoxic myopathy associated with atrial enlargement (24). Studies have shown that polygala tenuifolia extract (PTE) has anti-obesity activity, and its mechanism is related to the expression of genes involved in lipid and cholesterol metabolisms in the liver, such as IDI1 (25). Glycerol-3-phosphate acyltransferase mitochondrial (GPAM), also known as GPAT1, is a member of the GPAT gene family. Its protease is the first step in the biosynthesis of triglycerides and phospholipids (26). The expression of GPAM can induce the formation of at least 50% triglycerides in the liver and adipose tissue (27). GPAM can further affect the expression of mRNA of key enzymes in the second step of triglyceride synthesis to regulate lipid metabolism (28). In contrast, the expression of GPAM is consistent with the results of the gene chip, but there was no statistically significant. CF may be related to the expression of genes related to glycolysis and cholesterol metabolism in the liver, and the effect on triglyceride metabolism may not be the main way to improve hyperlipidemia.

Compared with the MC, 68 genes were both significantly enriched in GO and KEGG analyses in the luteolin intervention group. The results of gene verification are consistent with the trend of gene chip. Acsl3 and Lpin1 were significantly downregulated, and Cyp7a1 was significantly upregulated. Cholesterol 7 α-hydroxylase (CYP7A) catalyzes the synthesis of bile acid in the liver and maintains cholesterol balance in the body. Studies have shown that the upregulation of its expression can promote the conversion of cholesterol into bile acid (29, 30). This is consistent with the results of our previous study that the enzyme activity in the liver of rats in the luteolin group was significantly increased, indicating that luteolin can promote the expression of CYP7A1 and thus activate its enzyme activity to promote the conversion of bile acids and exert a hypolipidemic effect. Long-chain fatty acid (ACSL) acyl-coA synthase (FAs) plays an important role in lipid biosynthesis, in which ACSL3 activity is thought to be related to adipocyte differentiation, and ACSL3 overexpression can promote the increase of lipid drop triglyceride content (31). TNF-α increased ACSL3 expression to induce lipid droplet formation in human endothelial cells (32). The red raspberry extract (RRE) can significantly reduce the level of blood lipid in mice with hyperlipidemia, and Acsl3 is one of the regulatory genes, accelerating the conversion of triglyceride to fatty acid (33). The results of our preliminary animal experimental study showed that the metabolic enzyme activity of triglycerides was significantly higher and fatty acid synthase activity was significantly higher in the luteolin group than the intervention effect of luteoloside, which may be related to the regulatory genes for the conversion of triglycerides into fatty acids. GPAM expression was downregulated in the luteolin intervention group, but the difference was not statistically significant. The hypolipidemic effect of luteolin may be related to the expression of genes related to cholesterol biosynthesis and transformation in the liver. In addition, affecting the differentiation of adipocytes may also be a way to improve hyperlipidemia.

Compared with the MC, 51 genes were both significantly enriched in GO and KEGG analyses in the luteoloside intervention group. Lpin1 was significantly downregulated, and Acaca and Cyp7a1 were significantly upregulated. The expression of Angptl4 was downregulated but the difference was not statistically significant. Acetyl CoA carboxylase (ACACA) is a key gene for *de novo* synthesis of fatty acids, downregulation of its expression can deeply inhibit the biosynthesis of fatty acids, and its expression is significantly increased in high-carbohydrate and high-fat diet rats (34). It plays an important role in anti-obesity and improves the pathogenesis of NAFLD by micronutrients or traditional Chinese medicine (35, 36). Studies on the anti-obesity mechanism of naringin and its glycoside showed that naringin can inhibit lipid accumulation and TG content in 3T3-L1 cells by regulating some genes related to lipid metabolism, including Acaca (37). The results of our previous animal experimental study showed significant differences in triglyceride metabolism-related enzyme activities in the luteoloside intervention group, which may be related to its regulation of gene expression including Acaca to reduce triglyceride accumulation in hepatocytes. In addition, CYP7A1 overexpression in the luteoloside intervention group was consistent with a significant increase in its enzymatic activity in the liver tissue. Angiopoietin-like protein 4 (Angptl4) is a key gene in the regulation of lipid and glucose metabolism (38), and hyperlipidemia levels were significantly reduced in Angptl4 knockout mice (39). The results of this study showed that compared with the control group, the expression of Angptl4 in liver tissues was downregulated after luteoloside intervention, but the difference was not statistically significant, indicating that Angptl4 may not be a key gene in the mechanism of luteolin lowering blood lipid.

The results of our previous *in vivo* studies showed that both CF and its main components, luteolin and luteoloside, have significant hypolipidemic and hepatic steatosis effects, but CFs seem to have stronger antioxidant and lipid metabolism-related enzyme activities, which may have better effects in the long run (16). In fact, the results of this study indicate that the hypolipidemic mechanism of CF is more complex, involving various biological processes such as cholesterol, fatty acid metabolism, and glycolysis. The hypolipidemic effect of luteolin and luteoloside is mainly through the synthesis and transport of cholesterol and the fatty acid metabolic pathway, suggesting that there may be other major components involved in the hypolipidemic mechanism of CF.

## Conclusion

The mechanism of CF to improve hyperlipidemia is very complex, mainly involving biological processes such as cholesterol and fatty acid metabolism and glycolysis, including 41 differential genes such as Sqle, Gck, and Idi1. Luteolin mainly involves the synthesis and transport of cholesterol, which may include 68 differential genes such as Acsl3, Cyp7a1, and Lpin1. Luteoloside mainly involves fatty acid metabolism, which may include 51 differential genes such as Acaca, Cyp7a1, and Lpin1. The functional pathways of CF and its main components, luteolin and luteoloside, may not be completely the same, and further study is needed on the mechanism of action of other components.

## Data availability statement

The original contributions presented in this study are included in the article/supplementary materials, further inquiries can be directed to the corresponding author.

## Ethics statement

This animal study was reviewed and approved by the Animal Ethics Committee, Southeast University.

## Author contributions

JS and ZW: experiment and thesis writing. CL: data analysis. HX and SW: scheme design. LY: quality control. GS: overall coordination. All authors contributed to the article and approved the submitted version.

## References

[1] Xiao C Dash S Morgantini C Hegele RA Lewis GF. Pharmacological targeting of the atherogenic dyslipidemia complex: the next frontier in CVD prevention beyond lowering LDL Cholesterol. *Diabetes.* (2016) 65:1767–78. 10.2337/db16-0046 27329952

[2] Karr S. Epidemiology and management of hyperlipidemia. *Am J Manag Care.* (2017) 23(9 Suppl.):S139–48. 28978219

[3] Liu H Jiao J Zhu M Wu X Chen W. Cross-cultural adaptation and validation of the FRAIL-NH scale for Chinese nursing home residents: a methodological and cross-sectional study. *Int J Nurs Stud.* (2022) 128:104097. 34649713 10.1016/j.ijnurstu.2021.104097

[4] Li D Zhang Y Liu Y Sun R Xia M. Purified anthocyanin supplementation reduces dyslipidemia, enhances antioxidant capacity, and prevents insulin resistance in diabetic patients. *J Nutr.* (2015) 145:742–8. 10.3945/jn.114.205674 25833778

[5] Hooper L Kay C Abdelhamid A Kroon PA Cohn JS Rimm EB Effects of chocolate, cocoa, and flavan-3-ols on cardiovascular health: a systematic review and meta-analysis of randomized trials. *Am J Clin Nutr.* (2012) 95:740–51. 10.3945/ajcn.111.023457 22301923

[6] Curtis PJ Sampson M Potter J Dhatariya K Kroon PA Cassidy A. Chronic ingestion of flavan-3-ols and isoflavones improves insulin sensitivity and lipoprotein status and attenuates estimated 10-year CVD risk in medicated postmenopausal women with type 2 diabetes: a 1-year, double-blind, randomized, controlled trial. *Diabetes Care.* (2012) 35:226–32. 10.2337/dc11-1443 22250063 PMC3263874

[7] Liang Y Chen J Zuo Y Ma KY Jiang Y Huang Y Blueberry anthocyanins at doses of 0.5 and 1 % lowered plasma cholesterol by increasing fecal excretion of acidic and neutral sterols in hamsters fed a cholesterol-enriched diet. *Eur J Nutr.* (2013) 52:869–75. 10.1007/s00394-012-0393-6 22684634

[8] Friedrich M Petzke KJ Raederstorff D Wolfram S Klaus S. Acute effects of epigallocatechin gallate from green tea on oxidation and tissue incorporation of dietary lipids in mice fed a high-fat diet. *Int J Obes.* (2012) 36:735–43. 10.1038/ijo.2011.136 21750518

[9] Nambiar DK Deep G Singh RP Agarwal C Agarwal R. Silibinin inhibits aberrant lipid metabolism, proliferation and emergence of androgen-independence in prostate cancer cells via primarily targeting the sterol response element binding protein 1. *Oncotarget.* (2014) 5:10017–33. 10.18632/oncotarget.2488 25294820 PMC4259402

[10] Baselga-Escudero L Pascual-Serrano A Ribas-Latre A Casanova E Salvadó MJ Arola L Long-term supplementation with a low dose of proanthocyanidins normalized liver miR-33a and miR-122 levels in high-fat diet-induced obese rats. *Nutr Res.* (2015) 35:337–45. 10.1016/j.nutres.2015.02.008 25769350

[11] Cui Y Wang X Xue J Liu J Xie M. *Chrysanthemum morifolium* extract attenuates high-fat milk-induced fatty liver through peroxisome proliferator-activated receptor α-mediated mechanism in mice. *Nutr Res.* (2014) 34:268–75. 10.1016/j.nutres.2013.12.010 24655494

[12] Lee JH Moon JM Kim YH Lee B Choi SY Song BJ Effect of enzymatic treatment of *Chrysanthemum indicum Linné* extracts on lipid accumulation and adipogenesis in high-fat-diet-induced obese male mice. *Nutrients.* (2019) 11:269. 10.3390/nu11020269 30691060 PMC6412706

[13] Kim WJ Yu HS Bae WY Ko KY Chang KH Lee NK *Chrysanthemum indicum* suppresses adipogenesis by inhibiting mitotic clonal expansion in 3T3-L1 preadipocytes. *J Food Biochem.* (2021) 45:e13896. 10.1111/jfbc.13896 34368979

[14] Lee MS Kim Y. *Chrysanthemum morifolium* flower extract inhibits adipogenesis of 3T3-L1 cells via AMPK/SIRT1 pathway activation. *Nutrients.* (2020) 12:2726. 10.3390/nu12092726 32899992 PMC7551773

[15] Nepali S Cha JY Ki HH Lee HY Kim YH Kim DK *Chrysanthemum indicum* inhibits adipogenesis and activates the AMPK pathway in high-fat-diet-induced obese mice. *Am J Chin Med.* (2018) 46:119–36. 10.1142/S0192415X18500076 29298511

[16] Sun J Wang Z Chen L Sun G. Hypolipidemic effects and preliminary mechanism of *Chrysanthemum* flavonoids, its main components luteolin and luteoloside in hyperlipidemia rats. *Antioxidants.* (2021) 10:1309. 10.3390/antiox10081309 34439559 PMC8389196

[17] Livak KJ Schmittgen TD. Analysis of relative gene expression data using real-time quantitative PCR and the 2(-Delta Delta C(T)) Method. *Methods* (2001) 25:402–8. 10.1006/meth.2001.1262 11846609

[18] Liu D Wong CC Zhou Y Cai Z Huang ZP Hu P Squalene epoxidase induces nonalcoholic steatohepatitis via binding to carbonic anhydrase III and is a therapeutic target. *Gastroenterology.* (2021) 16:2467–82.e3. 10.1053/j.gastro.2021.02.051 33647280

[19] Sun H Li L Li W Yang F Zhang Z Liu Z P53 transcriptionally regulates SQLE to repress cholesterol synthesis and tumor growth. *EMBO Rep.* (2021) 22:e52537. 10.15252/embr.202152537 34459531 PMC8490977

[20] Mir MM Mir R Alghamdi MAA Wani JI Elfaki I Sabah ZU Potential impact of GCK, MIR-196A-2 and MIR-423 gene abnormalities on the development and progression of type 2 diabetes mellitus in Asir and Tabuk regions of Saudi Arabia. *Mol Med Rep.* (2022) 25:162. 10.3892/mmr.2022.12675 35293603 PMC8941532

[21] She L Li W Guo Y Zhou J Liu J Zheng W Association of glucokinase gene and glucokinase regulatory protein gene polymorphisms with gestational diabetes mellitus: a case-control study. *Gene.* (2022) 824:146378. 10.1016/j.gene.2022.146378 35276241

[22] Qin S He Z Wu Y Zeng C Zheng Z Zhang H Instant dark tea alleviates hyperlipidaemia in high-fat diet-fed rat: from molecular evidence to redox balance and beyond. *Front Nutr.* (2022) 9:819980. 10.3389/fnut.2022.819980. 35223953 PMC8875000

[23] Nakamura K Mori F Tanji K Miki Y Yamada M Kakita A Isopentenyl diphosphate isomerase, a cholesterol synthesizing enzyme, is localized in Lewy bodies. *Neuropathology.* (2015) 35:432–40. 10.1111/neup.12204 25950736

[24] Fang CY Chen MC Chang TH Wu CC Chang JP Huang HD Idi1 and Hmgcs2 Are Affected by Stretch in HL-1 atrial myocytes. *Int J Mol Sci.* (2018) 19:4094. 10.3390/ijms19124094 30567295 PMC6321625

[25] Wang CC Yen JH Cheng YC Lin CY Hsieh CT Gau RJ *Polygala tenuifolia* extract inhibits lipid accumulation in 3T3-L1 adipocytes and high-fat diet-induced obese mouse model and affects hepatic transcriptome and gut microbiota profiles. *Food Nutr Res.* (2017) 61:1379861. 10.1080/16546628.2017.1379861 29056891 PMC5642193

[26] Brockmöller SF Bucher E Müller BM Budczies J Hilvo M Griffin JL Integration of metabolomics and expression of glycerol-3-phosphate acyltransferase (GPAM) in breast cancer-link to patient survival, hormone receptor status, and metabolic profiling. *J Proteome Res.* (2012) 11:850–60. 10.1021/pr200685r 22070544

[27] Chen YQ Kuo MS Li S Bui HH Peake DA Sanders PE AGPAT6 is a novel microsomal glycerol-3-phosphate acyltransferase. *J Biol Chem.* (2008) 283:10048–57. 10.1074/jbc.M708151200 18238778 PMC2442282

[28] Yu H Zhao Z Yu X Li J Lu C Yang R. Bovine lipid metabolism related gene GPAM: molecular characterization, function identification, and association analysis with fat deposition traits. *Gene.* (2017) 609:9–18. 10.1016/j.gene.2017.01.031 28131819

[29] Pizzini A Lunger L Demetz E Hilbe R Weiss G Ebenbichler C The role of Omega-3 fatty acids in reverse cholesterol transport: a review. *Nutrients.* (2017) 9:1099. 10.3390/nu9101099 28984832 PMC5691715

[30] Olkkonen VM. Role of microRNA-185 in the FoxO1-CYP7A1 mediated regulation of bile acid and cholesterol metabolism: a novel target for drug discovery? *Atherosclerosis.* (2022) 348:53–5. 10.1016/j.atherosclerosis.2022.03.023 35397875

[31] Lv Y Cao Y Gao Y Yun J Yu Y Zhang L Effect of ACSL3 expression levels on preadipocyte differentiation in Chinese red steppe cattle. *DNA Cell Biol.* (2019) 38:945–54. 10.1089/dna.2018.4443 31355674

[32] Jung HS Shimizu-Albergine M Shen X Kramer F Shao D Vivekanandan-Giri A TNF-α induces acyl-CoA synthetase 3 to promote lipid droplet formation in human endothelial cells. *J Lipid Res.* (2020) 61:33–44. 10.1194/jlr.RA119000256 31722970 PMC6939593

[33] Tu L Sun H Tang M Zhao J Zhang Z Sun X Red raspberry extract (*Rubus idaeus L shrub*) intake ameliorates hyperlipidemia in HFD-induced mice through PPAR signaling pathway. *Food Chem Toxicol.* (2019) 133:110796. 10.1016/j.fct.2019.110796 31472226

[34] Dankel SN Bjørndal B Lindquist C Grinna ML Rossmann CR Bohov P Hepatic energy metabolism underlying differential lipidomic responses to high-carbohydrate and high-fat diets in male wistar rats. *J Nutr.* (2021) 151:2610–21. 10.1093/jn/nxab178 34132338 PMC8417924

[35] Khatiwada S Lecomte V Fenech MF Morris MJ Maloney CA. Effects of micronutrient supplementation on glucose and hepatic lipid metabolism in a rat model of diet induced obesity. *Cells.* (2021) 10:1751. 10.3390/cells10071751 34359921 PMC8304500

[36] Li X Ge J Li Y Cai Y Zheng Q Huang N Integrative lipidomic and transcriptomic study unravels the therapeutic effects of saikosaponins A and D on non-alcoholic fatty liver disease. *Acta Pharm Sin B.* (2021) 11:3527–41. 10.1016/j.apsb.2021.03.018 34900534 PMC8642447

[37] Dayarathne LA Ranaweera SS Natraj P Rajan P Lee YJ Han CH. Restoration of the adipogenic gene expression by naringenin and naringin in 3T3-L1 adipocytes. *J Vet Sci.* (2021) 22:e55. 10.4142/jvs.2021.22.e55 34313040 PMC8318791

[38] Xiao S Nai-Dong W Jin-Xiang Y Long T Xiu-Rong L Hong G ANGPTL4 regulate glutamine metabolism and fatty acid oxidation in nonsmall cell lung cancer cells. *J Cell Mol Med.* (2022) 26:1876–85. 10.1111/jcmm.16879 35285130 PMC8980907

[39] Li Y Gong W Liu J Chen X Suo Y Yang H Angiopoietin-like protein 4 promotes hyperlipidemia-induced renal injury by down-regulating the expression of ACTN4. *Biochem Biophys Res Commun.* (2022) 595:69–75. 10.1016/j.bbrc.2022.01.061 35101665

